# Exploring Tertiary Health Science Student Willingness or Resistance to Cultural Competency and Safety Pedagogy

**DOI:** 10.3390/ijerph18179184

**Published:** 2021-08-31

**Authors:** Sowbhagya Micheal, Anita Eseosa Ogbeide, Amit Arora, Stewart Alford, Rubab Firdaus, David Lim, Tinashe Dune

**Affiliations:** 1 School of Medicine, Western Sydney University, Campbelltown Campus, Locked Bag 1797, Penrith, NSW 2751, Australia; 2 School of Health Sciences, Western Sydney University, Campbelltown Campus, Locked Bag 1797, Penrith, NSW 2751, Australia; a.ogbeide@westernsydney.edu.au (A.E.O.); a.arora@westernsydney.edu.au (A.A.); stewart.alford@westernsydney.edu.au (S.A.); r.firdaus@westernsydney.edu.au (R.F.); david.lim@westernsydney.edu.au (D.L.); t.dune@westernsydney.edu.au (T.D.); 3 Health Equity Laboratory, Campbelltown, NSW 2560, Australia; 4 Translational Health Research Institute, Western Sydney University, Locked Bag 1797, Penrith, NSW 2751, Australia; 5 Discipline of Child and Adolescent Health, Sydney Medical School, Faculty of Medicine and Health, The University of Sydney, Westmead, NSW 2145, Australia; 6 Oral Health Services, Sydney Local Health District and Sydney Dental Hospital, NSW Health, Surry Hills, NSW 2010, Australia

**Keywords:** cultural competency and safety, teaching and learning, student transition, student retention, patient-centered care

## Abstract

There is an increasing body of literature that considers the relevance and experiences of cultural competency and safety training in health professional students. However, less is written about Australian tertiary learners’ experiences of engaging with cultural competency training. The aim of this study is to explore tertiary students’ willingness or resistance to cultural competency and safety pedagogy. Qualitative student feedback to a teaching unit was collected and triangulated with data from focus groups with tutors. Results were thematically analyzed. Willingness and resistance to cultural competency and safety teaching emerged as two key themes. Willingness to engage with the unit was largely due to student interest in the content, teaching environment and relevance of cultural competency to students’ future practice. Resistance was linked to the students feeling personally attacked, or culturally confronted, with tutors noting the topics around sexuality and white privilege being more resisted. Acknowledging reasons for student resistance and developing strategies to reduce resistance can facilitate more student engagement with cultural competency topics, ultimately leading to their future provision of culturally competent healthcare.

## 1. Introduction

Increasing population diversity creates challenges and opportunities for health professionals to care for culturally and linguistically diverse patients. Research indicates that poorer health outcomes may result if sociocultural differences between patients and health professionals are not reconciled [1,2,3]. Widespread racial and ethnic disparities in healthcare have been an impetus for the promotion of cultural competence in health settings [4]. Cultural competence is a key strategy to provide quality care that meets the social, cultural and linguistic needs of diverse patients [1]. The goal of cultural competence in healthcare is to create health systems and workforce that can deliver quality care to patients regardless of race, ethnicity, culture or language proficiency [1]. Cultural competence has been widely written about and defined [5], and most definitions are a combination of defining attributes of cultural competency, including cultural awareness, understanding, knowledge, sensitivity and skills [6]. Cross et al. [7] define cultural competency as a set of “congruent behaviors, attitudes and policies that come together in a system, agency or among professionals and enable that system, agency or those professionals to work effectively in cross-cultural situations”. A culturally competent system is one that values diversity, has capacity for cultural self-assessment, is conscious of inherent dynamics between cultures, has institutionalized cultural knowledge and adapts to diversity of its members [7]. Cultural competence also includes organizational and individual knowledge, conviction and capacity to take action [8]. Cultural competence in healthcare settings can improve patient health through integration of culture and acknowledgement of diversity in health service delivery [6,9].

There is agreement that consequences of cultural competence are positive, and the concept is worth promoting, particularly in communities where healthcare professionals of dominant cultures provide care for patients from minority cultural, social, linguistic, sexual or religious groups, including from disadvantaged or marginalized communities [10]. Cultural competency promotes interpersonal skills for health professionals to interact effectively with people from diverse backgrounds (colleagues and patients), thus improving patient health and wellbeing [11]. Culturally competent healthcare has been shown to positively impact patients’ perceptions of quality healthcare [12], enhanced care-seeking and treatment adherence [13], satisfaction with health providers [14], effective communication and interaction between patient and healthcare providers [1] and improved health outcomes [15]. 

First proposed in New Zealand in the 1990s, cultural safety focuses on healthcare delivery through reflections on power relationships and patient rights [16]. Cultural safety requires health professionals to reflect on societal power differentials and their own interpersonal power differences with their patients while providing healthcare, particularly for marginalized and vulnerable communities [16]. Cultural safety facilitates health practitioners to reflect on their own realities and the attitudes they bring to caring for their patients and be open-minded about people who are different from themselves [17]. Cultural safety promotes the development of a cadre of self-aware health professionals who practice culturally safe healthcare as defined by the people they serve [17] and continuously self-evaluate to ensure they focus on their patients without being influenced by assumptions about patients’ cultural or socioeconomic backgrounds [18]. In countries with colonial histories, culturally safe healthcare assists in altering the colonial relationships and creates safe spaces for Indigenous people within the healthcare system [19], allowing them to reshape the system [18].

Acknowledging the positive consequences, cultural competency and safety training has been incorporated in many universities’ health professional courses and plays a key role in students’ understanding of diversity [20]. Cultural competency pedagogy enables students to look beyond personal, organizational, professional and systemic ethnocentrism [21]. Students are encouraged to develop a cultural relativism mindset that allows them to acknowledge patient individuality, and empathize and understand patients’ actions through the lens of cultural norms and values [22]. Paving a way for future health professionals to be more self-aware and to reflect on their constructions about others’ lives is core to the teaching of cultural competence and safety [11,16]. The primary goal of cultural competency training for health professional students is to improve the health and wellbeing outcomes of students’ future patients by continually improving the practitioner–patient therapeutic alliance. Cultural safety pedagogy focuses on historical, social, political and economic circumstances that create inequalities in health, particularly for Indigenous communities [17], and promotes students’ continuous self-reflection [18]. Cultural competency and safety training is critical to build a cadre of culturally competent healthcare professionals who are sensitive to patients’ contexts and needs and are also open to the fact that cultural competence and safety is a lifelong endeavor and not a destination, requiring a lifelong dedication to self-criticism and reflection [23].

Many studies have been conducted to evaluate the impact of culture competency teaching and learning [24,25]. However, there is a surprising dearth of evidence in this area in Australia, with the majority rightfully being in relation to Aboriginal communities. While existing research provides an understanding of the potential for cultural competency training in Australia, there is little knowledge about how cultural competency and safety teaching more broadly is experienced by Australian learners. Learner experiences in other countries fall into broad categories of willingness and resistance [26]. Willingness to engage with cultural competency training can be influenced by learners’ pre-existing attitudes, timing, context and content of the curriculum [27]. Resistance is an important barrier to cultural competency training [24]. Reasons for resistance amongst students include student apathy [28,29], lack of culturally competent role models [30] and viewing cultural competence as a less valuable ‘soft science’ when compared to clinical knowledge [31]. Resistance can manifest as harm–loss appraisals, beliefs rationalizing the appraisals, negative emotions and defensive coping mechanisms [32]. Training on cultural competence and safety aims to assist students in gaining an appreciation of how diversity is viewed as a result of the social, political and cultural norms and standards that shape the experience of health in Australia [33]. Culturally competent and safe healthcare is particularly important in a culturally diverse country like Australia, where 2.8% of the population are Indigenous, more than 300 languages are spoken and more than one-third of the population is overseas-born [34]. Western Sydney University caters to the underserved communities of Greater Western Sydney, one of the most diverse and fastest growing areas in Australia, with over 50% of the population being migrants or descendants from more than 170 countries [34]. The aim of this study is to explore students’ engagement with cultural competency and safety pedagogy in the Culture Diversity and Health (CDH) unit, using student and tutor perspectives on the learning and teaching experiences. 

### Culture Diversity and Health Unit

Following the principles of Engaged Pedagogy and Performative Teaching [35], the CDH course, a core unit that discusses cultural competency and cultural safety for health professional students (undertaking clinical or allied health courses such as sports and exercise science, paramedicine, podiatry, occupational therapy and speech pathology), encourages students to be involved in real-world issues within their own identifiable cultural contexts and promotes cultivating active citizenship amongst students. The CDH unit runs over a 14-week semester through 13 non-compulsory lectures and 13 non-compulsory tutorials. Reflecting the diverse student population and acknowledging the needs of underserved and marginalized communities of Greater Western Sydney, the subject teaches social, cultural and sexual diversity and their applicability to health professional practice and patient-centered care. Indigenous Australia is an important topic to promote students’ appreciation of the needs and achievements of Indigenous people. Healthcare experiences of Culturally and Linguistically Diverse (CALD) Australians is another key topic. The importance of human rights and health equity on health outcomes of marginalized communities is highlighted in the unit. Additionally, the unit focuses on the impact of gender, ageing and disability on health outcomes. Strategies, best practices, frameworks and reflective practice to cultivate culturally safe and competent health practice are also discussed in the unit. Each year, approximately 750 students from the multiple health disciplines enroll in the CDH unit. Enrolled students include those just out of high school and mature age students who have completed other degrees and/or worked for a number of years. 

Using the findings, strategies to teach cultural competency and safety in a sensitive manner to improve willingness to learn cultural competency will be explored. The study explores the research question: what does health professional students’ feedback to teaching in the CDH unit indicate about their willingness and/or resistance to cultural competency teaching and learning?

## 2. Materials and Methods

### 2.1. Research Design

The study utilizes an explorative qualitative design using data from Student Feedback on the Unit (SFU), Student Feedback on Teaching (SFT) and focus group discussions with tutors of the CDH unit. Purposive homogenous sampling [36] of students and tutors involved in the course from 2013 to 2020 allowed for an in-depth exploration of the research questions [37]. The SFU and SFT surveys are routinely administered by Western Sydney University for all enrolled students at the end of the semester and participation in the feedback process is voluntary. Qualitative data were sought through two open-ended questions: best aspects of the course/teaching and areas for improvement. The University provides anonymized summaries of the SFU data to unit Coordinators and SFT data directly to tutors (unit Coordinators do not receive student feedback to tutors). The anonymous surveys provide opportunities for students to provide qualitative reflections without fear of repercussions. 

### 2.2. Data Collection

SFU data for the period of 2013–2020 were received from 1529 of the total 5242 students enrolled in the course for the 2013–2020 period (response rate of 29%). All tutors who taught in the course during the same period were invited to share their SFTs and participate in focus groups to explore their perceptions of students’ willingness and/or resistance to cultural competency learning. Ten tutors provided their SFTs for the study. These SFTs included feedback from a total of 1342 students. Three focus groups of approximately 90 min were held with a total of eight tutors between September 2020 and February 2021. Focus groups were moderated by author S.M., who has never taught in the CDH unit. Due to the restrictions placed by the COVID-19 pandemic, focus groups were held via Zoom. Focus groups were audio-recorded and participants provided verbal consent on record. Tutors’ names were redacted, pseudonyms assigned and SFTs de-identified prior to data analysis. 

### 2.3. Data Analysis

Qualitative data from the SFUs and SFTs were collected and thematically analyzed. Focus group data were transcribed and thematically analyzed using a deductive approach [38]. All focus group audio recordings were transcribed by author A.E.O. and checked for accuracy. Using a deductive approach, analysis started with the initial broad categories of willingness and resistance to cultural competency training that emerged from existing literature. Authors A.E.O., T.D. and S.M. individually familiarized themselves with the data and assigned preliminary codes. The coding process was then discussed before reaching a consensus on final codes and themes. Emerging and substantive categories within the participants’ statements were defined in relation to the objectives of the analysis. Analysis focused on topical responses and coding for phrase repetition, clear and emotional comments, as well as dialogue markers. 

## 3. Results

Results presented here capture students’ and tutors’ views on student engagement in the CDH unit. Preconceived themes of willingness and resistance to cultural competency and safety teaching and learning emerged as key themes from the SFU, SFT and focus group data (Table 1). 

### 3.1. Willingness to Engage in Cultural Competency Training

Examples of willingness and resistance to cultural competency and safety teaching and learning were described by both students and tutors. Tutors discussed seeing examples of students’ willingness and resistance to their cultural competency and safety teaching.

“*I got everything from absolutely a sort of door slammed shut, lack of willingness* [to stay in the room] *to a really sincere, open and kind of warm willingness to learn about material that these* [students] *had never really considered, so there was both* [resistance and willingness]*.*” (Sean, CDH tutor for 1 year)

Student feedback indicated that their willingness to engage in the CDH unit was linked to their perception of unit tutors, understanding of the course content and lived experiences, as discussed below in Section 3.1.1, Section 3.1.2, Section 3.1.3.

#### 3.1.1. Student Perceptions of the Tutor 

Willingness to engage in the teaching and learning activities were linked to tutors’ ability to create a fun, safe and engaging learning environment. Students remarked that “*the tutors interest boosted engagement and participation*” and that “*tutor creates an informal learning environment which works*”. Students perceived more freedom to express their thoughts on cultural competency in an honest way due to the tutors’ ability to create an open and non-judgmental learning environment. This impacted students’ willingness to attend non-compulsory tutorials with some students being “*excited to attend tutorials*”.

The tutors’ ability to discuss the course content and unpack students’ thoughts on cultural competency was cited by many students. Students identified that “*tutors provided guidance to help understand the content of the unit*” and “*the way the unit was taught provided genuine interest*” for the students to engage. Students viewed the tutors as being “*culturally competent and empathetic towards students*”, which makes the tutors have the ability to “*be willing to elaborate*” on questions asked as well as being “*open to new ideas*”. 

Students also described that tutors’ teaching style and use of multiple teaching resources allowed them to comprehend and analyze the course topics better. For example, students found the use of multiple types of teaching resources to be an effective way to engage with the unit content.

“*YouTube series made it a lot easier to learn the content by having smaller doses at one time, made it easier to soak in the information.*”

Students also mentioned how a tutor style of “*mixing banter with learning pushes for everyone to get involved*”*,* while using “*personal life examples*” in explaining a context or a phenomenon in their “*current society*” got the students “*to challenge their beliefs with others within a safe environment*”.

#### 3.1.2. Applicability of Course Content to Students’ Personal and Professional Lives 

The topics taught in the unit captured students’ interests and promoted student engagement, participation and reflection on contemporary cultural issues. Some students perceived the unit as relevant to their lives and professional careers.

“*I appreciate that* [the unit] *gave me an insight into other cultures and an in-depth understanding of topics such as racism, I felt I was blinded to.*”

Another student expressed that CDH was a “*thought provoking unit*” and “*the assessment task equipped students with skills to be job ready, shaping an ethical and professional future work force*” which “*exposes the purpose of what it means to be culturally competent*”.

Tutors also expressed that students’ ability to apply their cultural competency and safety training to their future health professional practice resulted in willingness to engage and participate.

“**[students in certain degree programs] *they sort of saw* [the unit] *as valuable because they felt that as first responders that they were going to be out and they would be interacting with different communities, different ethnicities, all those different range of issues, more so than what other professions potentially would in their professional life. So, for them, they saw the value of being involved in that tutorial.*” (Zeke, CDH tutor for 4 years)

Tutors also expressed that students’ perceptions of the applicability of cultural competency in their personal and professional lives was often linked to students’ own lived experiences.

#### 3.1.3. Lived Experiences of Students 

Belonging to an ethnic minority and mature age while enrolled in the unit emerged as key features that increased willingness to engage with unit content. Some students appreciated the unit’s teaching content as it resonated with their own lived experiences of belonging to a non-dominant culture.

“*Having heard off-handish comments* [about my culture] *from people not only in society but from students, I can strongly see the need for this subject. This unit enables students to step out of their own skin and start to realize the barriers that other people in the community have to go through. As up and coming health professionals, it seems this* [unit] *is so important … I have thoroughly enjoyed this unit.*”

Tutors pointed out that some students “*want to know more … more information, links, books and lectures*” and “*engage with the content*” than other students. Relatability to unit content as a result of lived experiences fostered willingness to engage.

[For] *the mature age students or students from a non-Anglo background, they’re the ones who have lived the experience or witnessed it with their grandparents or their parents and the issues that they face if they don’t speak English well or racism or, you know, they’ve already had some experience and they may not have experienced it themselves yet or visibly, but they’re aware of it, obviously, for Aboriginal students as well. And maybe Anglo women would come next because they’re aware of this, bit of a generalization, but more aware of, you know, the sort of imbalance of power for men and women. And everyone can see that in their own families and, you know, in the generational story.*” (Lily, CDH tutor for 1 year)

Age of the student, assessed by tutors during tutorial discussions, was highlighted as an important factor that influenced willingness to engage. Mature age students were observed to participate more in CDH discussions.

“*Mature age student’s kind of appreciate and understand what we are talking about culture, diversity and health and all the equity and inequality and social justice.*” (Mia, CDH tutor for 1 year)

Despite students and tutors identifying precursors and clear examples of student willingness to engage with cultural competency and safety pedagogy, data also indicated strong resistance amongst students to the unit’s content.

### 3.2. Resistance to Cultural Competency Training

Students discussed reluctance to engage with the unit due to “*feelings of fear, confrontation, disrespect or anger*”. Tutors observed resistance to discuss key unit content and students’ inability to question their own beliefs. 

#### 3.2.1. “Attacking” Dominant Cultures

Some students expressed feelings of being confronted by the course content which they perceived as focused on attacking privileged, dominant cultures.

“**[The course] *content was portraying hatred against* [people] *who were from healthy backgrounds*”

“*The tutor seemed to hold a grudge against white people* [and the content] *came across racist, invalid and untrue*”.

On the contrary, tutors agreed that it was not necessarily the unit content that led to resistance. Rather, it was students’ reflexivity and ability or lack thereof to question their own individual beliefs and ideology.

“**[It is] *the subject matter* [not necessarily] *about cultural competency, but the ideas surrounding race, gender and sexuality* [which are] *core components of identity.*” (Andrew, CDH tutor for 5 years)

Tutors gave examples of their experiences of teaching about dominant and minority cultures, and students’ resistance to this teaching.

“*When I was talking about privilege or white privilege, it was students who were open* [and] *willing to listen to what exactly we mean when we talk about white privilege. And there were other students, who were like no, I don’t believe* [privilege or white privilege] *exist.*” (Mia, CDH tutor for 1 year)

Tutor and student data indicated strong student resistance to engaging with topics of white privilege and Australia’s colonial history. Such resistance often manifested as students feeling personally confronted and offended.

#### 3.2.2. Students Feeling Personally Attacked

Students expressed feelings of being personally attacked during in-class discussions and fear of being targeted due to belonging to a majority cultural group.

“*As a white/Anglo individual, I felt the topics were controversial which spilled hypocrisy*”.

Another student commented that “*every white person in this unit were wildly offended*” due to the “*white shaming*” content which “*led to decisions not to attend classes*”. Students described feelings of being “*responsible and guilty for historical events*”.

Tutors described that students who felt attacked often did not want to engage in tutorial discussions mainly because they were confronted by hearing thoughts and opinions different from their own.

“**[students not] *having to kind of think through that sort of content and sort of think through those sorts of cognitive dissonance moments and kind of going, I don’t want to think about this. So, I think I’d rather just kind of not be there and just get through the assessments and get on with it …* [the unit] *requires some thinking of maybe suspending my own beliefs for a session for a second and actually thinking about other people. And I think for some people, that kind of often in a very disruptive, controversial way in the tutorials when people would express those different things and having those conversations, some people just don’t want to engage in that.*” (Zeke, CDH tutor for 4 years)

#### 3.2.3. Questioning Relevance of Cultural Competency and Safety Teaching

Student perceptions on the CDH unit’s utility to their future practice influenced their engagement with cultural competency teaching and learning, with some students stating that the course was pointless.

“*The course was common sense and basic knowledge which should not be taught.*”

Some students expressed that the unit was “*partial*” to certain cultures and did not find the content relevant to their future health professional practice. Tutor data also indicated that at times, students made public displays of resisting cultural competency teaching, discussed below. 

#### 3.2.4. Resistance in Action

Student resistance to engage with the CDH unit content manifested as students leaving group discussions mid-way and non-completion of the unit. Students who appeared personally attacked by content attempted to create discontent amongst their classmates instead of engaging and reflecting on teaching topics.

“*The instigator of a different opinion, who* [sometimes] *wants to stretch* [discussion] *of a* [teaching topic] *to a breaking point, to kind of maybe push buttons or otherwise.*” (Zeke, CDH tutor for 4 years)

Tutors noted that some students were confronted by specific topics such as sexuality and Aboriginal history.

“*Just in terms of the course content, it starts with Aboriginal history, and so I think it’s because … it’s quite confronting for a lot of people. And I say more, students in the class have never thought about the concept of or understand Aboriginal, the history of Aboriginal Australia and the racism and intergenerational trauma and all the rest of it that they have to face every day. That’s very confronting for a lot of students.*” (Lily, CDH tutor for 1 year)

“**[Discussing] *some elements, particularly homosexuality, was a big one and gender fluidity, race, racial and cultural differences* [which] *people were more into. But it was the sexual stuff that seemed to knock* [students] *off balance.*” (Sean, CDH tutor for 1 year)

Zara, a CDH tutor for 8 years, commented that “*halfway through* [the unit] *or a third way through where* [students] *are forced to a reckoning,* [during] *the lectures, students walk out* [and] *walked on*”.

#### 3.2.5. ‘Type’ of Students

Three ‘types’ of students were described—those that are very enthusiastic about the unit at the outset, those who would like to “*just get over*” with the unit to complete their course requirements and those who enter the unit with a negative outlook about unit content.

“*Those students who see the class as a requirement and they are just trying to get it over with. The other category is, students who view the class as an interesting one and lastly, students who come to the class actively hostile because they have ideological issues with the course content.*” (Andrew, CDH tutor for 5 years)

Tutors agreed that students who enter the CDH unit with an open mind engaged well with the content as opposed to students who were unwilling to think broadly about unit topics.

“*Students who come in very open-minded to these different ideas* [in the unit] *that kind of contradicts with what they already know are probably the one who get that transformative experience throughout the course … And if there’s someone who is just really, I guess, closed and very dogmatic, it’s going to be hard to crack them no matter how much we* [try] *it.*” (Mia, CDH tutor for 1 year)

The ‘type’ of student was found to impact their levels of engagement in the unit, with tutor feedback indicating the open-minded, older and ethnic minority students being most likely to engage.

### 3.3. Recommendations to Improve Student Willingness

Tutors provided recommendations to improve future students’ willingness to engage with cultural competency and safety teaching in the CDH unit (Table 2).

## 4. Discussion

This qualitative study provides evidence of tertiary health professional students’ experiences of cultural competency and safety teaching and learning at an Australian university. Findings from this study provide additional insights into learner willingness or resistance to cultural competency training. Results from the study indicated examples of resistance to cultural competency and safety training amongst health professional students. A key finding from this study is that students’ resistance is linked to their willingness to explore new thoughts and ideas about their own and others’ cultures. Students were particularly resistant to topics such as white privilege and sexuality, and found it difficult to engage with discussions on these topics. Resistance to teaching about white privilege has been previously identified [39,40,41]. Despite the resistance, teaching white privilege is critical to improve students’ understanding of colonial histories, systemic oppression of certain communities and to raise awareness about their roles and responsibilities with diverse future patients [40]. Similar to this study’s findings, resistance to teaching on gender and sexuality in multicultural education courses has been reported internationally [42,43]. Demonstrating genuine concern for student resistance and discomfort [41], asking students how they want to handle challenging topics at the beginning of the semester and seeking mid-semester feedback to determine how students are coping with discussing topics such as white privilege and sexuality have been proposed [42]. Inviting and listening to guest speakers from culturally diverse backgrounds [42] also improves students’ understanding of people’s lived realities.

This study found that students’ open-mindedness to learn about cultural competency increased their willingness to engage with cultural competency teaching. Henderson et al. [6] cite six key antecedents to cultural competence, including (1) openness to learning about new cultures, arising from a flexible attitude and willingness to reflect on one’s own ethno-culture, beliefs and behaviors, (2) motivation to want to be more knowledgeable, skillful and aware of others’ cultures and (3) cultural sensitivity, a cognitive and affective component that involves attitudes, perceptions and values that illustrate awareness of one’s own culture and recognition and respect for others’ cultures. Students’ resistance may be due to their lack of these critical antecedents to cultural competence and not necessarily due to their objections to the course content. Further research is needed to explore CDH students’ antecedents to cultural competence and determine any links between these antecedents and resistance to cultural competency training. 

According to Wear and Aultman [44] and Mackean et al. [45], resistance may take the form of challenging course materials and teaching staff when aspects of inequality are presented as systemic inequality or power differentials. This literature is supported by study findings of students expressing resistance through challenging course content and walking out of teaching sessions. Study findings also support existing literature that shows that there are discernible patterns of resistant student behavior, ranging from negative emotions, defensive coping mechanisms and students appraising teaching topics as harmful [32,46]. Resistance can be the result of feeling confronted by values and concepts that are different to those students have been brought up with. Opposition can also result from students failing to see the significance of cultural competency and safety training in their future careers and lives [47]. Examples of resistance found in this study are of particular interest as research demonstrates that students who are resistant to cultural competency and safety teaching are less likely to engage with the content or to enact the principles of cultural competency [48,49]. Long term, such resistance precludes the development of therapeutic relationships with future patients [26], which can negatively influence the healthcare system in a multicultural country like Australia. 

Strategies to improve students’ engagement with cultural competency and safety topics is crucial to facilitate students’ future culturally competent and safe health practice, particularly in their care for marginalized and underserved communities. Students reported greater willingness to engage with cultural competency teaching due to tutor engagement, creation of a safe learning environment and teaching style. Novel methods of engaging students are needed to reduce resistance and increase their involvement in the ongoing journey of cultural competence [50]. Cultural competence pedagogy entails delivering rigorous curriculum to students, designing engaging teaching and learning experiences and creating practical evaluations [51]. Promoting pathways for cultural competency learning that allows for multi-perspective, collaborative and peer-learning opportunities with simultaneous experiential tasks promotes cultural competency learning [52]. Tutors’ ability to ask pertinent questions that encourage students to unpack their assumptions and allow them to openly discuss concepts such as systemic inequity [53,54], racism and historic oppression may improve student willingness to engage with these topics. Strategies to improve willingness can also include involving students in cultural competency curriculum development [55,56]. Some authors argue that culturally competent practitioners must move beyond cultural competency learning and self-reflection to experiential learning in order to build skills in working with multicultural communities [57,58]. Study results indicate that willingness to engage with cultural competency training was fostered when students found unit topics relevant to their professional practice. Immersive learning experiences where students encounter patients from cultural backgrounds different from their own may be a strategy to emphasize the relevance of cultural competency in future practice and improve sensitivity to other cultures. In addition, as suggested by tutors, progression of multiple courses that discuss cultural competency may ensure that students revisit topics at different stages of health professional degrees instead of engaging with the topics for a single unit of study. Future teaching in the CDH unit will be improved using tutor recommendations, study findings and existing literature. 

Results showed that mature age students tended to be more willing to engage with cultural competency topics as opposed to younger high school leavers. Willingness of mature age students to engage in cultural competency training may be attributed to their lived experiences. Resistance of younger students may be a consequence of student transition from educational environments that do not regularly challenge their values to the same level that tertiary cultural competence training does. A better ability to support student transition to tertiary education may result in effective engagement in cultural competency training, which is inherently designed to promote the development and maintenance of a positive therapeutic alliance between health practitioners and their patients [59]. Including mature age students at the end of their degrees as role models or ‘champions’ of cultural competence to encourage junior students to engage in cultural competency and safety training [28] may be useful. Additionally, further research is required to determine any impact of current or recent local, national or international sociopolitical events on younger students’ resistance to engage with cultural competency training. 

A limitation of this study is that it captures students’ experiences in only one unit, which may not be generalizable to different student populations. Future research with students through interviews or focus groups can allow a better understanding of resistance and strategies to increase willingness. The data collected also do not consider the influence of student diversity on their willingness or resistance to cultural competency training. Abreu et al. [60] suggest that multiculturalism in an academic program is impacted by student composition, with at least 30% non-dominant group representation required. Further research into the influence of classroom diversity on students’ resistance and willingness to cultural competency, particularly in hyper-diverse areas such as Greater Western Sydney, would be useful. 

## 5. Conclusions

Students in the CDH unit reported willingness and resistance to cultural competence teaching and learning. Resistance included feeling attacked by course content and questioning the relevancy of cultural competency topics to future practice. Despite evidence of resistance to unit contents, students also expressed desire to engage with teaching topics in a safe learning environment created by tutors. While the impact of cultural competency and safety training may presently not be perceived as valuable or relevant, as indicated by students, it is significant that the learning process still takes place to allow for the student to gradually unravel the cultural awareness construct [61] that allows for self-reflection of personal biases and stereotypes towards culture, race, sexuality and health. Students may continue to unpack cultural competence constructs [61] over time, which may be influenced by lived experiences, future professional experience or desire to engage in culturally competent and safe healthcare.

## Figures and Tables

**Table 1:** Themes and sub-themes emerging from data.

Themes	Sub-Themes
Willingness	Student perceptions of tutor Applicability of course content to students’ personal and professional lives Lived experiences of students
Resistance	“Attacking” dominant cultures Students feeling personally attacked Questioning relevance of cultural competency and safety training Resistance in action ‘Type’ of students
Recommendations to Improve Student Willingness	Highlight relevance of future practice Supplementary unit in later years Build trust between students and tutors

**Table 2:** Recommendations from tutors.

Recommendation	Supporting Quote
**Highlighting relevance to future practice:** Continuously linking cultural competency topics to students’ future practice as health professionals may improve engagement with these topics.	“*Trying to link to their profession is always something I found was helpful. I guess the best strategy is kind of making it relevant to them and where they’re at, what they’re doing.*” (Zeke, CDH tutor for 4 years)
**Supplementary unit in later years of the students’ course:** The CDH unit could be an introduction to culture and diversity. Developing another unit towards the end of students’ degree may enable more reflection and engagement with cultural competency teaching topics.	“*There maybe needs to be another, for students who are doing* [this] *course in first year of university or somewhere in the second year. Gee, maybe there needs to be another course like that in the later years so that they’ve actually got a chance to absorb it and to look around them and think about it and come back to it for another round and develop that way of thinking.*” (Lily, CDH tutor for 1 year)
**Building trust between students and tutors:** Building trust with students, through open discussions, can improve willingness to engage with unit content.	“*Right at the very beginning about this being an open and trusting and trustworthy environment, because I had a sense that there was going to be, you know, a fair bit of disagreement turned around and challenged and I think it worked.*” (Sean, CDH tutor for 1 year)

## Data Availability

The data are not publicly available.
